# Rationale and Feasibility of Resistance Training in hEDS/HSD: A Narrative Review

**DOI:** 10.3390/jfmk7030061

**Published:** 2022-08-20

**Authors:** Hannah A. Zabriskie

**Affiliations:** Department of Kinesiology, Towson University, Towson, MD 21252, USA; hzabriskie@towson.edu

**Keywords:** hypermobility, strength training, safety, Ehlers–Danlos syndrome, exercise prescription

## Abstract

Hypermobile Ehlers–Danlos Syndrome (hEDS) and hypermobility spectrum disorder (HSD) are genetic conditions characterized by increased joint hypermobility, often in the presence of other signs or symptoms if syndromic. This hypermobility can result in significant pain and ultimately decreased participation in recreational or competitive activity. Rehabilitation of patients with hEDS/HSD is not well understood, particularly since presentation can be relatively heterogenous. Regardless, more research is needed, particularly regarding resistance training, to allow patients with hEDS/HSD to participate in the activities they enjoy. The purpose of this narrative review is to provide an overview of the clinical features displayed by those with hEDS/HSD that have been found to be improved with resistance training in other populations, and to present the current evidence for resistance training in all types of study designs, ranging from case studies to randomized controlled trials.

## 1. Introduction and Rationale

### 1.1. Background, Features, and Habits of hEDS/HSD

In the latter half of the 20th century, clinicians began to describe a number of connective tissue disorders in parallel: Ehlers–Danlos Syndrome (EDS) Type III [1] and Benign Joint Hypermobility Syndrome (BJHS) [2]. Both conditions have had a number of different aliases in the intervening years, though as the medical literature on this topic has grown, experts have suggested these two conditions are not two separate entities, but rather two conditions on the same clinical spectrum [3]. This spectrum of disease, formally known as Hypermobility Spectrum Disorder (HSD), ranges from benign generalized joint hypermobility (GJH) without symptoms to patients with significant symptoms and/or serious disablement meeting the most recent, and stringent, diagnostic criteria for hypermobile EDS (hEDS) [4]. Because of the spectral nature of these conditions and the small amount of literature on this topic, the general term “hEDS/HSD” will be used throughout this review, and research described herein includes populations described as having any of the following: BJHS, EDS Type III, EDS Hypermobile type (EDS-HT), Joint Hypermobility Syndrome (JHS), GJH, hEDS, and/or HSD. Throughout, the diagnostic term used by the original author will be employed.

It is not uncommon for patients with hEDS/HSD to demonstrate a number of features that, theoretically, can be improved with resistance training or other exercises. For instance, resistance training has been shown to increase muscular strength, [5,6,7,8,9,10,11,12,13], muscular endurance [7,11,12], and muscular cross-sectional area (CSA) [9,13,14,15,16] in a wide variety of populations. Further, bone mineral density (BMD) is improved [17,18,19,20,21,22] and body composition is enhanced with gains in lean muscle and decreases in body fat [16,23,24,25,26] when participants regularly perform resistance training across the lifespan. Using body weight movements, evidence has shown that balance [5,27,28,29,30,31,32,33,34,35,36,37,38] can also be improved through exercise. Each of these attributes may be diminished in individuals with hEDS/HSD, though not necessarily consistently, and this evidence is described hereafter.

### 1.2. Musculoskeletal Features

Aside from the hallmark joint laxity that defines hEDS/HSD, there are additional musculoskeletal features that may benefit from exercise training.

#### 1.2.1. Bone

There is a substantial amount of data regarding bone features in individuals with EDS, primarily bone mineral density (BMD). However, it is not uncommon for reports to combine results for multiple types of EDS, particularly hypermobility and classical types [39,40,41]. Further, there are sometimes conflicting results, particularly between studies that state that they exclusively studied BJHS and those who enrolled patients with a type of EDS. Dual-Energy X-Ray Absorptiometry (DXA), X-ray, and peripheral quantified computed tomography (pQCT) are common methods used in these studies.

With regards to cohorts exclusively consisting of BJHS participants, there are equivocal results. A 1996 investigation by Mishra et al. [42] suggested that there is no statistical difference in lumbar or femoral neck BMD between BJHS patients and the normal population. However, when specifically evaluating individuals less than 45 years of age (*n* = 26), BMD trended towards being poorer (Lumbar: *Z* = −0.297; Femur: *Z* = −0.401), though the significance threshold was not reached. Mishra et al. did include males in their sample, though the entire sample did not undergo DXA assessment and there is no way to know how many men are included in those less than 45 years of age. A more recent report by Gulbahar and colleagues [43] noted that women with BJHS had significantly lower femoral neck, total femoral, and trochanteric BMD. Gulbahar et al. found that women with BJHS were 1.8 times more likely to have low bone mass than healthy controls. Of note, there was no difference in physical activity between the BJHS and control participants in the report from Gulbahar et al. [43], a variable not explored by Mishra et al. [42].

Reports who have studied cohorts composed of exclusively EDS patients (though often a combination of different EDS types, as noted earlier) have consistently found EDS patients to have poorer femoral neck BMD than healthy controls [39,40,41,44,45]. Lumbar spine BMD has been reported to be poorer in some cohorts [39,40], though not all [41,44,45]. One cohort composed exclusively of patients with EDS Type III was noted to have no differences in lumbar spine BMD compared to healthy controls despite poorer femoral neck BMD [44,45]. However, differences in femoral neck BMD disappeared after accounting for height, weight, and physical activity in EDS Type III patients. Mazziotti et al. [41], who studied a group of 52 individuals with classical or hypermobility EDS found no difference in lumbar or femoral neck BMD when compared to healthy controls when the two types of EDS were grouped together. When the two diagnoses were analyzed as separate groups, it was revealed that EDS-HT had poorer femoral neck BMD compared to both classical EDS and control subjects, while no differences were noted in lumbar spine. In addition to poorer BMD in some body regions, peripheral fracture incidence in EDS populations (namely, Type I, II, and III) has been reported to be as much as 10 times greater than healthy controls [39], and individuals with classical, hypermobile, or classic vascular-like EDS also experience vertebral fractures at a much higher rate [40,41].

Most recently, a report from Banica and colleagues [46] thoroughly investigated bone and muscle properties in a cohort of 43 people with hEDS/HSD. They found no differences in lumbar spine or whole-body BMD compared to healthy controls. However, given that a number of previous studies have similarly shown no differences in spine BMD but significant differences in the femoral neck, it is a shortcoming of this study that femoral neck values were not obtained. Using pQCT, Banica et al. determined that hEDS/HSD patients tend to have smaller cortical bone area, lower cortical bone mineral content, smaller cortical thickness, and decreased strength strain index in the lower leg, though no differences in trabecular or cortical volumetric BMD were noted. A higher major fracture incidence was also reported in the hEDS/HSD population. The authors suggest that the higher fracture rate is primarily due to decreased peak bone size and mass, likely secondary to lower amounts of physical activity during adolescence and young adulthood, as will be discussed in a later section. Banica and colleagues recommend resistance training to help combat some of these musculoskeletal findings, while noting that the ideal rehabilitation regimen for this population is still unknown. They likewise note that the stress applied to the skeleton during exercise that can trigger positive changes in bone must be of moderate–high intensity; this becomes very difficult to accomplish in hEDS/HSD as there are common injuries, symptoms, or kinesiophobia that may prevent completion of such exercise. There are very few papers utilizing a resistance training program in the hEDS/HSD that use greater resistance than body weight or elastic resistance bands, and that data will be covered in the second half of this review. The role of exercise in developing and maintaining a healthy bone mineral density and preventing osteopenia/osteoporosis is well documented and research is needed to identify how it can be safely implemented in the hEDS/HSD population.

#### 1.2.2. Muscles and Tendons

In the last 10 years, a number of reports describing muscle and tendon properties in exclusively hEDS/HSD populations have been published. These reports consistently document hEDS/EDS Type III/EDS-HT patients to have lower muscular strength [47,48,49,50,51], endurance [47,50,51], and functional performance [47,48] compared to controls. One study [49] separated EDS-HT and GJH patients and noted that EDS-HT patients had poorer lower body muscular strength than their hypermobile, but pain- and syndrome-free, counterparts with GJH. However, a recent study [51] comparing subjects with hEDS found no differences between those with hEDS and HSD with regards to muscle strength, though both groups were substantially impaired compared to healthy controls. In some cohorts, decreased strength remained significantly different from controls even after accounting for fat-free mass [48] or physical activity [47].

When it comes to quantifiable measures of the musculotendinous unit such as muscle cross-sectional area (CSA), there have been conflicting results from the same laboratory. In 2012, Rombaut et al. [52] reported that there were no differences in CSA of the lower leg between EDS-HT cases and healthy age-matched controls; however, in 2020, Banica et al. [46] reported that hEDS/HSD patients had smaller CSA of the lower leg even after accounting for confounding variables such as height, weight, and physical activity. The same laboratory has also provided evidence that hEDS/HSD individuals have lower passive muscle tension as well as lower Achilles tendon stiffness when compared to healthy controls [52].

Two studies have gone beyond measuring raw strength or muscle attributes to publish information that will greatly aid with practical application. First, Scheper et al. [48], using linear models, were able to determine that muscle weakness has a significant impact on activity limitations (i.e., poorer performance in a chair sit-to-stand task and the 6 min walk test (6MWT)) in EDS-HT. Further, the authors noted that proprioceptive ability confounded the role of muscle weakness, but only for the sit-to-stand task, not the 6MWT. This suggests that while muscle strengthening should be a key goal of exercise programs, proprioceptive gains or resistance training in the setting of proprioceptive challenge should likewise be considered an integral component of any program. Second, To and Alexander [49] were able to determine that individuals with EDS-HT, though having less muscular strength to begin with, can gain strength at a similar rate as control participants performing the same exercise intervention. Therefore, practitioners should expect patients and clients to make regular strength gains. The authors note that the change in muscular strength over the course of 12 weeks of their study was approximately 40%. Previous research has found that young adults without pain or hypermobility can experience strength gains much greater than 40% (upwards of 150%) over 12 weeks [13]. However, heavy loads (at least 60% of one-repetition maximum (1RM) for untrained individuals and 80%1RM for recreationally active individuals) should be used to maximize strength gains [53,54]; an intensity that has largely been deemed inappropriate in a population dealing with pain and hypermobility. Because of the risks associated with the determination of 1RM in patients with hypermobility, many studies do not perform this assessment and do not use %1RM values to determine exercise prescriptions, a common practice in healthy populations.

### 1.3. Decreased Proprioception and Balance

Rombaut et al. [55] conducted the first investigation into joint position sense in EDS Type III patients. The protocol required 32 females diagnosed with EDS Type III and 32 healthy controls to position their shoulder and knee joints at specific angles both passively and actively. Females with EDS Type III had significantly poorer joint position sense at the knee than their healthy counterparts, as indicated by higher passive (6.9° vs. 4.6° at 30° of flexion; 7.4° vs. 5.8° at 60° of flexion) and active (6.7° vs. 4.0° at 30° of flexion; 5.4° vs. 4.3° at 60° of flexion) absolute errors in joint angle. Altered joint position sense was later validated by Sahin and associates [56] in individuals with BJHS. In patients with hEDS/HSD, proprioceptive deficits such as altered joint position sense are likely contributors to pain, injuries, difficulty with complex movements, and the development of osteoarthritis [56].

Rombaut and colleagues [57] noted that, with regards to balance, women with EDS-HT have greater sway when standing on a flat surface or on a cushion both when eyes are open and closed compared to healthy controls. Individuals with EDS Type III also walked slower and with shorter step and stride lengths. Further, when EDS Type III patients were asked to complete a mathematical calculation in their heads while walking, their walking speed slowed even more, whereas healthy controls maintained the same speed. Celletti et al. [58] documented that the vertical component of ground reaction force decreased as self-reported fatigue increased in EDS-HT. Because fatigue is a common symptom in hEDS/HSD, many individuals may experience this type of gait pattern most of the time. Ground reaction force has been used as a diagnostic to identify pathologic gaits and may suggest decreased accuracy of proprioception [58]. The increased sway, division of attentional resources, and diminished ground reaction force may also predispose individuals with hEDS/HSD to injury, particularly when fatigue is elevated.

### 1.4. BMI and Body Composition

Though not the main purpose of any identified research study, a few papers have identified some concerning trends in body mass or composition when describing their hEDS/HSD populations. Higher BMI in EDS-HT compared to healthy controls has been noted by Rombaut et al. [57] (26 vs. 23 kg/m^2^, *p* = 0.004) and Scheper et al. [48] (27.8 vs. 22.8 kg/m^2^, *p* < 0.001). Most recently, Banica and colleagues noted that hEDS participants not only had higher BMI than control at baseline (*n* = 82, 27.5 vs. 24.3 kg/m^2^, *p* < 0.05) and at an eight-year follow-up with a subset of the original population (*n* = 44, 28.5 vs. 25.1 kg/m^2^, *p* < 0.05), but they likewise noted that hEDS participants had less lean mass (41.2 vs. 43.2 kg, *p* < 0.05) and greater fat mass (25.1 vs. 23.3 kg, *p* < 0.05) at baseline, as determined by DXA. While hEDS participants maintained a greater fat mass at the 8-year follow-up (25.6 vs. 23.4 kg, *p* < 0.05), they no longer had significantly less lean mass than their healthy counterparts (42.3 vs. 43.5 kg, *p* > 0.05). Though these studies did not specifically intend to assess body composition or weight concerns in the hEDS/HSD population and there is not enough evidence to definitively state that hEDS/HSD populations will have poorer body composition, the addition of resistance training to an exercise regimen would certainly help to address any compositional concerns in this population.

### 1.5. Physical Activity, Exercise Habits, and Physical Therapy Experiences in hEDS/HSD Populations

The relationship between a hypermobile phenotype and some of the features detailed above seems to be moderated by physical activity. Generally, hEDS/HSD populations tend to have a lower amount of participation in both sport and leisure time physical activity (PA) [46,50,59]. A questionnaire-based study [60] reported that 26.6% of hEDS/HSD patients participated in 60–150 min of exercise per week and an additional 26.2% of patients participated in more than 150 min per week. However, the intensity of the activity was not recorded.

A relatively high proportion of hEDS/HSD participate in regularly scheduled physical therapy compared to healthy controls (33.3% vs. 0%) [50], though they often report being treated dismissively by physical therapists, therapists not knowing how to treat this condition [60], or that therapy has a negative or neutral effect on their treatment outcomes [61]. Researchers concluded that patients with hEDS/HSD value physical therapists who work in partnership, communicate clearly, and are knowledgeable about the condition [60]. This underlies the importance of evidence-based practice in this population.

With regards to physical activity and exercise counseling, it is not uncommon for medical providers to tell patients with hEDS/HSD that they have no specific physical activity restrictions, “but if it hurts, don’t do it”. Over time, this may lead to increased removal of activities that promote health, fitness, and mental or emotional well-being without direction on how to return to the activity or how to prevent pain from the outset. Because of this, an unrestricted narrative review of the literature is warranted to further investigate current practices in treating hEDS/HSD. For this purpose, we aim to summarize the available literature and provide recommendations on general exercise prescription, clinical treatment, and future research.

## 2. Materials and Methods

Ending on 20 July 2022, PubMed, Google Scholar, and Ebsco Host were searched for published peer-reviewed literature using but not limited to the following keywords: hypermobility, Ehlers–Danlos syndrome, joint hypermobility, strength, resistance, training, exercise. When possible, effect sizes (Cohen’s d) were calculated for each statistically significant (*p* < 0.05) relevant outcome. However, due to small sample sizes and the use of non-parametric methods in many reports, it was not possible to calculate effect sizes for many reported outcomes. Due to these challenges and the inherent difficulty of comparing studies using slightly different participant populations and styles of intervention, summary tables were developed that outline the following: sample size, included diagnoses, exercise intervention, duration and frequency of intervention, and significant results.

## 3. Review of the Literature Using Resistance training in the hEDS/HSD Population

Little research has been conducted on the effect of resistance training in patients with hEDS/HSD and all previously published research has focused on training within the scope of rehabilitation and physical therapy, and no research to date has investigated resistance training for anything more than symptom management in this population. The purpose of this review is to provide an overview of research including strength or resistance training in the hEDS/HSD and to explore the rationale for recommending resistance training to this population. Future directions will likewise be provided.

An interesting aside—while three [62,63,64] of the presented case studies come from American authors, none of the original research or review articles that will henceforth be cited are from the United States of America, which may suggest an area for improvement and growth in education programs for exercise and rehabilitation professionals in the USA.

### 3.1. Case Studies

Though case studies have limited generalizability, it is still worthwhile to present the most relevant results from such publications. In 1986, Hinton [62] published what was likely one of the first case reports suggesting that an intensive exercise prescription may be beneficial for patients with hEDS. This report investigated the role of exercise in the care of a 10-year-old female who experienced frequent shoulder dislocations, patella and hip subluxations, and ankle instability and had been diagnosed with either EDS Type III or EDS VI (no clarification was provided as to whether genetic testing was pursued or not). See Table 1 for program details. After 5 weeks of training the patient was able to move through the entire shoulder ROM without pain and had no further subluxations and subsequently progressed to swimming, throwing, and other normal pursuits for a 10-year-old without incident, but with persistent ankle instability. It was reported that when the patient decreased adherence to the home exercise program nearly a year after initially seeking care, an increase in shoulder dislocations and ankle sprains occurred. Over 20 years later, Kitagawa et al. [65] published a similar report detailing the care of a 14-year-old female suffering from EDS-HT and recurrent shoulder dislocations. The patient completed a two-phase program, with each phase lasting 3 months (see Table 1 for sample exercises). Both phases were supplemented by home exercises. The program resulted in significantly increased shoulder stability and increased pain-free range of motion. Both of these case studies suggest that resistance training can be beneficial in acutely symptomatic adolescents with hEDS, though it is critical that adherence to the home exercise program be maintained to avoid a return of pain and dislocations.

In active adults, the course of care has changed over time. In 2000, Russek [64] published a case study on a 28-year-old very active JHS patient, who happened to be a physical therapist. The patient was counseled to decrease her level of activity and to specifically terminate participation in calisthenic strengthening exercises. The termination of a resistance training mode seems counterintuitive to management strategies for this condition, particularly since no other strengthening exercise was prescribed. Another case study published by Pennetti [63] provided further evidence for therapeutic exercise in an already active EDS-HT subject. The 35-year-old female previously completed triathlons and sought care after developing cervical and lumbar radiculitis and a history of many other musculoskeletal complaints. After 16 weeks of supervised treatment, supplemented with a home program, which focused on spinal, abdominal, cervical, and scapular stabilization exercise, the patient had pain-free ROM in both the cervical and lumbar spine and demonstrated increased strength (assessed via manual muscle testing) in the periscapular and hip muscle groups.

Zhou et al. [66] published a brief presentation of two EDS Type III cases, both adult females (23 and 41 years of age, respectively). While it was stated that exercise was prescribed to both patients, little detail was provided about the exercise program, which lasted 6–8 weeks. Both patients reported less pain following the intervention, though the role of exercise among the multitude of treatments employed is unclear (see Table 1).

In total, these cases suggest that a targeted exercise program can help to alleviate musculoskeletal dysfunction and pain at specific problem joints.

### 3.2. Studies in Children and Adolescents

A total of three trials using therapeutic exercise (including resistance training) have been conducted in children and adolescents aged 7–16 [67,68], and two of them were included in a systematic review by Peterson et al. [69] in 2018. None of the three studies utilized a true “control” group, though two studies compared different treatment approaches. Kemp et al. [68] investigated the effect of a generalized whole-body exercise program vs. a program targeted at symptomatic joints in 57 children and adolescents with BJHS. The intervention involved 6 weeks of a generalized or targeted supervised physical therapy program, supplemented by daily home exercises. The generalized program focused on maximizing general muscular fitness, whereas the targeted program focused on “controlling neutral” position of symptomatic joints both dynamically and at rest with a goal to establish greater motion and postural control (see Table 2 for sample exercises). The primary outcome in this investigation was pain, which decreased significantly after both programs and remained decreased an additional three months after termination of therapy. While baseline strength values are provided, strength was not reassessed during follow-up visits. However, results for the six-minute shuttle test were reported before and after the exercise intervention and no significant differences were noted between the treatment programs.

Pacey et al. [67] studied the effect of resistance training on pain at the knee joint in 25 children and adolescents with BJHS across 8 weeks of supervised therapy using one of two treatment paradigms: extending to a neutral position for all movements or hyper-extending for all movements. Both paradigms used the same movements and exercises, with the only difference between experimental groups being the prescribed range of motion at the knee joint. See Table 2 for included exercises. Pain, psychosocial functioning, thigh strength, and number of flights run in two minutes were assessed before and after the intervention. Again, pain was the primary outcome variable and pain decreased significantly in both groups after training concluded. An interesting finding was that parents tended to report better physical functioning in their affected child after exercise in the neutral position; however, parents reported greater positive changes in psychosocial behaviors following training with hyperextension, suggesting that utilizing the full ROM that hypermobile children experience and treating it as normal may help children and adolescents to feel more comfortable with their diagnosis. No group differences were reported for thigh strength or number of flights ran between the two interventions. However, when grouping all participants together there was a significant training effect with a statistically significant increase in thigh strength, as measured by a handheld dynamometer (Δ = +1.06 N, *p* = 0.004, *d* = 0.53).

In 2020, a report by Van Meulenbroek and colleagues [70] demonstrated that resistance training in addition to exposure therapy can increase muscle strength and decrease pain in adolescents with hEDS/HSD and kinesiophobia. While the details of the exercise intervention were not provided, the authors do report a significant improvement in knee extension strength and endurance as well as knee flexion strength and endurance, Additionally, the reported 63% decrease in pain scores is remarkable.

These studies suggest that not only are exercise interventions focused on increasing muscular fitness in children and adolescents with hEDS/HSD successful at managing the most troubling symptom of their condition (pain), but patients can also glean functional benefits because of training. While exercise certainly seems to be beneficial, there is still no consensus on the optimal mode, intensity, or progression of exercise to utilize in this population, particularly after a successful baseline rehabilitation program. Further, none of these studies utilized a control group. Ideally, healthy controls should be enrolled in the same or similar program to help identify if response to exercise is similar between children and adolescents with hEDS/HSD and those without, which should help to establish standards of care. Further, as discussed by Peterson et al. [69], neither the cohort presented by Kemp et al. [68] nor Pacey et al. [67] achieved their target sample sizes identified in their a priori power analysis, leaving both studies under powered.

The ultimate goal in strength and resistance training in an hEDS/HSD pediatric population should focus on managing symptoms and enabling safe play, particularly in sports when an injury may be more likely to occur. To date, research has focused on symptom management and no data is available describing interventions aimed at safe athletic performance. In a systematic review, Engelbert et al. [71] suggest using sports or hobbies of the patient’s preference to facilitate adherence to the treatment plan. However, they also note that many children and adolescents with hEDS/HSD may not be able to play their sport of choice simply because of their joint instability.

### 3.3. Studies in Adults

A total of ten investigations were identified in which some form of strength or resistance training was prescribed to an hEDS/HSD adult (minimum age of 16 years) population. Studies are grouped by the primary training goal: knee proprioception, lumbar stabilization, optimization of activities of daily living (ADLs), and improving strength. Only two studies used muscular strength as a primary outcome, though others did report metrics of muscular fitness in their findings. The final research study included in this review is a feasibility study investigating the use of a heavy-weight resistance training program in an hEDS/HSD population and will be highlighted separately.

#### 3.3.1. Primary Training Goal: Knee Proprioception

A number of investigations have focused on increasing the proprioception at the knee joint. The first of these reports was published in 2004 by Ferrell and colleagues [72] who investigated the role of closed kinetic chain (CKC) exercises on knee joint proprioception and balance when performed four times per week for eight weeks in 18 participants (16 females) with BJHS. See Table 3 for sample exercises and modes of resistance. Participants began the program with squats, pliés, and bridging while the other exercises were gradually added into the program along with increasing the number of sets and repetitions performed. The program was unsupervised, and patients reported their compliance. Results indicated a statistically significant change in threshold detection angle (Δ = −0.28°, *p* < 0.001, *d* = −4.9), which suggests a significant improvement in proprioception. Balance was likewise improved as the time spent out of balance decreased by 4.5% after the exercise program (*p* < 0.001, *d* = −3.6) as measured by an instrumented balance board. Significant improvements in peak and average torque for both hamstrings and quadriceps were recorded using an isokinetic dynamometer (*p* < 0.05).

Sahin et al. [56] also tested the ability of exercise to improve knee proprioception in 40 participants with BJHS. The exercise intervention was 8 weeks in length, similar to Ferrell et al. [72]; however, participants only trained on three days per week in a supervised clinic. The exercises involved in the program published by Sahin et al. [56] focused heavily on movements to augment balance (see Table 3 for sample exercises). The researchers utilized a hypermobile control group who did not undergo an exercise intervention. After eight weeks of exercise, BJHS subjects who completed the exercise intervention had significantly improved average absolute angle error values (AAAEV) in both right (*p* < 0.001, *d* = 1.8) and left (*p* < 0.001, *d* = 1.9) knees compared to controls. BJHS patients also demonstrated significantly improved AAAEV in both knees compared to baseline (Right knee: Δ = −0.9, *p* = < 0.001, *d* = −1.2; Left Knee: Δ = −0.8, *p* = 0.001, *d* = −1.2). Strength was not assessed in this experiment.

Most recently, Daman and associates [73] investigated whether an accelerated combination of the programs previously published by Sahin et al. [56] and Ferrell et al. [72] could improve knee joint proprioception in women with BJHS (N = 24, intervention = 12, control = 12). Instead of an eight-week intervention, participants were tested before and after four weeks of training (see Table 3 for program specifics). Further, Daman et al. [73] actively excluded any patient who reported exercising regularly (i.e., ≥ three times per week). A goniometer was used to assess proprioceptive error at the knee. In a non-weight bearing position, participants who completed the intervention had a significantly lower angle error than those in the control group (Δ = −3.0°, p = 0.009, *d* = -0.5), and compared to baseline (Δ = −1.8°, p = 0.01, *d* = −0.3). Similar results were noted in the weight-bearing position, with post-intervention errors being much less in the exercise group compared to control (Δ = 3.2°, *p* = 0.03, *d* = −0.4) and compared to their own baseline (Δ = −3.7°, *p* = 0.005, *d* = −0.4). Again, changes in muscular strength were not measured.

#### 3.3.2. Primary Training Goal: Lumbar Stabilization

Toprak-Celeny et al. [74] investigated the role of an 8-week lumbar stabilization training program in women with BJHS. A total of 38 women were randomized to either exercise intervention or control. The exercise intervention focused on teaching muscle activation and abdominal bracing, which progressed through static/postural stability, dynamic, and functional movements. See Table 3 for program details. Before and after the program muscular endurance of the trunk and postural stability were assessed using McGill’s isometric tests [75] and a Biodex Balance System, respectively. Significant increases in muscular endurance were identified for trunk flexion (Δ = 31.5 sec, *p* = 0.003), trunk extension (Δ = 32 sec, *p* < 0.001), as well as right (Δ = 28 sec, *p* = 0.001) and left (Δ = 26.5 sec, *p* < 0.001) side bridge. No changes in muscular endurance were identified in the control group.

#### 3.3.3. Primary Training Goal: Optimize Activities of Daily Living

Bathen et al. [76] took an interesting approach to improving ADLs by incorporating both physical training and cognitive–behavioral interventions into their training regimen, with the additional goal of decreasing kinesiophobia in their cohort of 12 women with EDS-HT or BJHS. The protocol consisted of 2.5 weeks of inpatient therapy and 3 months of home training, concluding with four days of inpatient assessment. During the baseline inpatient stay, all participants participated in lectures and discussions focused on developing tools to better manage pain and other symptoms during daily life and included topics such as sleep, nutrition, coping, and social networks. Participants were also introduced to resistance training during the baseline inpatient stay. The home exercise protocol can be found in Table 3 and was completed 5 days per week with 15–30 repetitions and 3 sets per exercise. Five of the twelve participants reported a clinically meaningful improvement in ADL performance and eight participants reported a clinically meaningful improvement in ADL satisfaction, as assessed by the Canadian Occupational Performance Measure. Participants demonstrated improved tandem walking backwards time (Δ = −9.05 sec, *p* = 0.006), walking up stairs (Δ = −0.13 sec, *p* = 0.004) and number of calf raising repetitions (Δ = +4.5 reps, *p* = 0.045) after completing the exercise intervention. Kinesiophobia also decreased significantly (*p* = 0.022). While many of these changes are statistically significant, the practical significance and feasibility of this program, particularly the inpatient component, is somewhat questionable. However, it does suggest that body-weight exercises are safe and reasonable in the hEDS/HSD population.

Recently, Reychler and colleagues [77] aimed to specifically train inspiratory muscle (IM) groups in female hEDS patients who demonstrated reduced IM strength (<80% predicted values). A total of 20 participants were enrolled and 10 participants completed a 6-week unsupervised IM training regimen involving 5 unsupervised training sessions per week consisting of 6 sets of 10 repetitions at increasingly higher resistance using an inspiratory muscle trainer. The other 10 participants served as control. While inspiratory muscles are not the typical muscle group one would think to conduct strength testing on, the authors did report maximal sniff nasal inspiratory pressure as a surrogate of muscular strength. Functional exercise capacity was also assessed using the 6MWT. Maximal sniff nasal inspiratory pressure increased significantly in the participants who completed the 6 weeks of training (Δ = + 8 cm H20, *p* = 0.003, d = 0.5) while no change was noted in those in the control group. Similarly, distance covered in the 6MWT increased significantly in the intervention group (Δ = 60 m, *p* = 0.036, d = 0.5). This demonstrates that even resistance training focused on a very small portion of the body can have a significant impact on ADLs in this population.

#### 3.3.4. Primary Training Goal: Gaining Muscular Strength

In perhaps one of the most relevant publications in relation to the purpose of this review, To and Alexander [49] published a report in 2019 detailing strength gains in individuals with JHS, GJH, and controls who went through a 16-week, personalized resistance training program. The program entailed training three times per week, and every other week participants met with a physical therapist who assessed muscular strength and developed appropriate exercise progression. Program information can be found in Table 3. As mentioned previously, JHS participants had significantly lower strength at the start of the protocol and required nearly the entire duration of the study to reach the baseline strength of individuals with GJH or controls. The authors note that the change in muscular strength over the course of 12 weeks of the study was approximately 40%. Previous research has found that young adults without pain or hypermobility can more than double their strength over 12 weeks [13]. The authors aptly note that to trigger higher gains of strength heavy or maximal loads should be used, which would be “inappropriate” in a population dealing with pain and hypermobility.

Recently, an investigation into a 12-week self-guided resistance training program in women with GJH was conducted (N = 51) [78]. Their cohort included women who were diagnosed or could have been diagnosed with hEDS/HSD (~43%) (see Table 3). After 12 weeks of the self-directed program, no changes in isometric knee extensor or flexor strength were identified; nor did muscle cross-sectional area or muscle mass increase. While it is discouraging that no improvements were identified over these three months, the authors posit a reason why this could have happened: a self-directed program may not have led to proper progression of the exercises. The authors note that the average leg press resistance for a single leg was 26 kg at the start of the 12 weeks and increased to 51 kg at the end of the intervention. While participants did double the amount of weight they were lifting, it was still only, on average, 83.5% of body weight. This suggests that participants may not have been progressively increasing their loads enough to develop improvements. Despite these results, or lack thereof, it is still encouraging that 63% of the training group completed at least 80% of the program. Given that this program appears to have included more strenuous movements than other studies, it is positive to note that exercising with resistance more than body weight without injury is possible in the hEDS/HSD population.

#### 3.3.5. Primary Research Goal: Feasibility of Heavy Shoulder Resistance Training Program in hEDS/HSD Populations

One of the most recent published reports [79] concerning resistance training in the hEDS/HSD population is a feasibility study assessing the implementation of a heavy shoulder strengthening exercise program in patients with pervasive shoulder symptoms. Liaghat et al. [79] implemented a 16-week program consisting of two supervised and one unsupervised exercise session each week. The program consisted of five open-chain, weighted exercises (see Table 3). During weeks 1–3, patients developed familiarity with the movements and performed three sets at 50–90% of their 10 RM. During weeks 4–9, this was progressed to 3 sets of 10 repetitions of their 10 RM and during weeks 10–15 patients performed 4 sets of their 8 RM. Week 16 was a taper week in preparation for post-program testing. Once the patient could perform more than the prescribed number of repetitions for every set without pain greater than 5/10 with “acceptable symptoms”, the weight was increased. This paradigm almost runs contrary to what practitioners may tell patients with hEDS/HSD concerning activities (i.e., “if it hurts, don’t do it”) and needs to be investigated more.

Promising results were obtained from this inquiry with regards to adherence, retention, and adverse events. Nine of twelve participants had 100% exercise adherence. Four participants reported minor adverse events (primarily soreness), which were deemed expected or unrelated to the intervention. Additionally, isometric shoulder strength improved between 28 and 31%, movement depending. Positive changes in joint laxity/instability and proprioception were also noted. Overall, this report suggests that not only is heavy resistance training effective in this population, but it is feasible without significant injury or symptoms. This suggests that heavy-weight open-chain kinetic exercises may be possible in individuals with hEDS/HSD.

## 4. Conclusions and Future Directions

In total, these studies suggest that resistance training is not only possible but effective in the hEDS/HSD population for managing symptoms and improving musculoskeletal function and fitness. In all ages of hEDS/HSD populations, the primary goals should be strength and stability. However, the current research does not yet provide enough evidence to define a standard rehabilitation or training paradigm for use in these populations or in preparation for specific activities. A summary of future research directions is provided in Table 4.

In children and adolescents, additional research is needed using control subjects to better understand the relationship between muscular fitness improvements and hypermobility. As suggested by Engelbert et al. [78], it would be ideal if the management plan paralleled a sport or activity of interest; however, identifying such an activity that does not cause exacerbations of joint pain or instability can be challenging in the hEDS/HSD population. In this age group, the aim of any training intervention should be increasing strength while minimizing pain and laxity, though additional research is needed to identify the efficacy of strengthening exercise in preventing injuries sustained during competitive or casual play.

In adults, there is evidence suggesting that patients with hEDS/HSD can benefit in a variety of ways from a structured strength intervention. Most interventions [49,56,70,71,72,73,75] have focused on closed kinetic chain movements using body weight, resistance bands, or balance boards as the primary form of resistance or challenge. While these styles of training are certainly safer for individuals with joint instability, there is also an inherent training ceiling with these modes. Nevertheless, the studies explored previously have shown that training in these modes can lead to decreased pain, increased ability to perform ADLs, increased stabilization, and increased muscular strength. Despite these results, several practical questions remain:

1. What should a rehabilitation program look like for the hEDS/HSD population and what is the proper progression, both regionally (e.g., training muscle groups in a specific order) and with regards to intensity/mode, to employ in such settings?
2. Can an hEDS/HSD patient who has successfully completed a supervised rehabilitation program and no longer experiences chronic dislocations, subluxations, or excessive pain safely progress to unsupervised resistance training in a gym setting? How?
3. Most research has been reactive in nature (i.e., training was prescribed in response to symptoms)—is there a proactive exercise program that could be given to patients as soon as they are diagnosed with hEDS/HSD in an attempt to “get in front of” injuries or symptoms?

In general, individuals with hEDS/HSD are encouraged to follow physical activity guidelines from the ACSM and AHA [78]. However, we echo the conclusions of several meta-analyses [78,79,80] that more research is needed to understand what the proper exercise prescription looks like in individuals with hEDS/HSD both in rehabilitative and supervised settings, and in an unsupervised setting. To allow for better generalizability, it is imperative that researchers carefully detail the contents of the intervention program, including specific movements, intensity, and progression, as well as employ a well-rounded fitness assessment both pre- and post-intervention. Further, it is common for investigations in this population to focus on rehabilitation or improvement of one specific joint, such as the knee. It is certainly helpful to have research performed on these frequently problematic body regions; however, a greater volume of research is needed for both specific regions and the whole body.

One relationship that has not been well elucidated is the impact of underlying fitness level on the outcomes of these strengthening interventions. While the hEDS/HSD population does tend to be less active [46,50,59,60], there are certainly active individuals with hEDS/HSD such as the subject of the case study by Penneti [64] who competed in a triathlon. It is likely that the previously explored studies, therefore, primarily included people who were relatively sedentary. In fact, Daman et al. [75] and Luder et al. [77] purposefully excluded anyone who participated in regular physical activity. This relationship cannot be overlooked in future research, as a baseline level of aerobic fitness may modulate the response to strengthening activities. Further, more active individuals in the hEDS/HSD population may need to develop strength and stability just as much as an inactive individual, they just experience less pain or better manage the symptoms of their syndrome, and therefore seek out rehabilitative help less frequently.

In conclusion, more research is needed to understand how to safely develop strength and stability in hEDS/HSD patients. It is important for healthcare providers to know safe modes, proper progression, and the potential ability of this patient population. As easy as it is to tell people with joint hypermobility to follow ACSM activity guidelines, it may be very challenging for these individuals to safely perform the recommended amount of exercise without a better understanding of how to protect themselves from injury. Patients with hEDS/HSD are often counseled to only perform movements that do not cause pain and for some this may mean giving up a sport or exercise mode that they enjoy. Often, they are not given guidance on returning to their sport or activity, rather they are simply told to cease doing it. However, there is not sufficient research to state that it is impossible for these individuals to return to their sport or activity after completing a proper strength and conditioning program. Additionally, there must be more research on resistance training in the hEDS/HSD populations using modalities other than resistance bands or body weight. The results from Liaghat et al. [76] are certainly promising as they utilized a more strenuous training stimulus with high levels of adherence; however, more research is warranted.

## Figures and Tables

**Table 1:** Outcomes of Case Studies including Exercise Advice or Prescription.

Study	Subject	Diagnosis	Intervention Goal	Intervention	Mode of Resistance	Duration; Frequency	Results
Hinton, 1986 [62]	10-year-old female	Primary: EDS type III or type XI	Increase strength, coordination, proprioception	Shoulder ab/adduction, internal/external rotation, and horizontal ab/adduction Shoulder press/lat pull down Bench press/shoulder retraction Hip ab/adduction Isometric eccentric exercises (all affected joints) Neuromuscular training (all affected joints)	Isokinetic dynamometer, weight machines, balance devices, body weight	11 weeks; 1–3×/week 11 months later returned to 2×/week for 4 weeks	At 5 weeks: Pain-free range of motion (shoulders) No subluxations At 10 weeks: ↑ strength in all muscle groups At 13 weeks: Return to play and normal activities Continued ankle instability At 1 year: ↔ strength (from 10 weeks) ↑ school attendance ↑ peer interaction
Russek, 2000 [63]	28-year-old female	Primary: Hyper-mobility syndrome	Manage pain and return to physically active lifestyle	Reduce “excessive” exercise Eliminate wrist weights during jogging Eliminate or limit calisthenics or martial arts Use protective and supportive splints *(no exercise intervention was administered)*	*NA*	Follow-up at 1 month and 1 year	At 1 month: ↓ pain Returned to jogging and martial arts At 12 months: ↓ pain frequency ↔ pain Decreased jogging No calisthenics Significant joint pain in new locations
Pennetti, 2018 [64]	35-year-old female	Primary: hEDS w/TNXB gene mutation Secondary: Cervical and lumbar radiculitis	Manage pain; return to physically active lifestyle	Postural reeducation Proprioceptive neuromuscular facilitation (PNF) of the scapula Myofascial trigger point release for lumbar spine and pelvis Spinal mobilization Core stabilization	Body weight, *otherwise not specified*	14 months; 2×/week for 16 weeks; 1×/week thereafter	Pain-free cervical and lumbar AROM ↑ periscapular strength ↑ hip strength ↑ neck flexor endurance ↓ pain (NPRS) ↑ function (PSFS)
Zhou et al., 2018 (Case 1) [65]	41-year-old female	Primary: hEDS	Manage chronic pain	Medication Coping strategies Education on postural awareness and body mechanics Kinesiotaping instruction “Exercise prescription with graded exercises, including pool activity.”	*Not specified*	2 months	↓ pain intensity after 2 and 18 months ↑ ADL ability after 2 and 18 months
Zhou et al., 2018 (Case 2) [65]	23-year-old female	Primary: EDS (type unspecified, assumed hEDS)	Manage chronic pain	Medication Coping strategies Education on symptom control and exercise program Relaxation techniques	*Not specified*	*Not Specified*	↓ pain
Kitagawa et al., 2020 [66]	14-year-old Female	Primary: hEDS Secondary: MDI of the shoulder	Improve scapular motor control and decrease MDI	Months 1–3: Isometric Movements Shoulder abduction In/external rotation Extension, and flexion Months 4–6: The Watson Program for MDI	Resistance band	6 Months; 1–2×/week	↑ Active Flexion ↑ Active Abduction ↑ Stability @ 6 mo. ↓ Stability @ 12 mo. after discharge to home program ↑ Motion ↑ Function Positive Sulcus sign @ 6 and 12 months

JHS = Joint Hypermobility Syndrome; BJHS = Benign Joint Hypermobility Syndrome; hEDS = Hypermobility Ehlers–Danlos Syndrome; HSD = Hypermobility Spectrum Disorder; NA = not applicable; ↑ = statistically significant increase was noted; ↓ = statistically significant decrease was noted; ↔ = no change was noted.

**Table 2:** Outcomes of Exercise Intervention Studies in Children and Adolescents with hEDS/HSD.

Study	N (% Female)	Comparison Group (*n*)	Age (Years)	Inclusion Criteria	Target	Mode of Resistance	Duration; Frequency	Results
Kemp et al., 2010 [67]	57 (33%)	Yes (27, generalized program)	10.9 (2.5)	Primary: BJHS	LE	*Not specified*	8 weeks; 6 total sessions Home program 7×/week throughout	At 8 weeks: ↓ pain ↓ parental assessment of pain ↓ Global score (Targeted only) ↔ 6 min shuttle test At 5 months: ↓ pain ↓ parental assessment of pain (Targeted only) ↓ Global score (Targeted only)
Pacey et al., 2013 [68]	26 (69%)	Yes (14, neutral ROM)	12.0 (2.9)	Primary: JHS Secondary: Knee pain	LE	Body weight, resistance bands	8 weeks; 2×/week for 4 weeks, once per two weeks thereafter Home program 5×/week throughout	Both Groups: ↑ thigh strength ↓ knee pain ↑ parent-reported physical and psychosocial summary scores ↔ stair ascent ↔ CHAQ functional measures Hyperextension ROM Group: ↑ CHQ psychosocial score ↑ self-esteem ↑ mental health Neutral ROM Group: ↑ physical summary score
Van Meulenbroek et al., 2020 [69]	14	None	17.5 (16.0–20.3) *	Primary: hEDS/HSD Secondary: Kinesiophobia	Core LE	*Not specified*	15 weeks total (8 weeks of exercise intervention followed by 5 weeks of exposure therapy)	↓ pain ↑ functional ability ↑ muscle strength ↑ motor performance

Age is reported as Mean (SD). * Data only reported as Median (IQR). JHS = Joint Hypermobility Syndrome; BJHS = Benign Joint Hypermobility Syndrome; hEDS = Hypermobility Ehlers–Danlos Syndrome; HSD = Hypermobility Spectrum Disorder; LE = Lower Extremity; MSK = Musculoskeletal; ROM = Range of Motion; UE = Upper Extremity; WB = Whole Body; ↑ = statistically significant increase was noted; ↓ = statistically significant decrease was noted; ↔ = no change was noted.

**Table 3:** Outcomes of Studies involving Exercise Prescription in Adults with hEDS/HSD.

Study	Total N (% Female)	Control Group *(n)*	Age (years)	Inclusion Criteria	Target	Mode of Resistance	Duration; Frequency	Results
Ferrell et al., 2004 [70]	18 (89%)	No	27.3 (10.4)	Primary: JHS Secondary: knee pain	LE	Body weight, balance board	8 weeks; 4×/week	↑ proprioception ↑ balance ↑ peak and avg quadricep strength ↑ peak and avg hamstring strength
Sahin et al., 2008 [56]	40 (73%)	Yes (25)	26.9 (7.2)	Primary: BJHS Secondary: knee pain	LE	Body weight, Balance board, mini-trampoline	8 weeks; 3×/week	↓ joint angle error ↑ occupational activity (AIMS-2)
Bathen et al., 2013 [71]	12 (100%)	No	35 *	Primary: EDS-HT/JHS	WB	Body weight, Resistance bands, Exercise ball	12 weeks; 5×/week	↓ tandem walking backwards time ↓ stair walking up time ↑ calf raise performance ↔ pain
Palmer et al., 2016 [72]	18 (94.7%)	Yes (Advice Only, 7)	33.5 (7.4)	Primary: JHS Secondary: no other conditions causing MSK pain	WB	Resistance band, body weight, ankle weights	16 weeks; 6 supervised sessions	↓ pain MDHAQ vs. advice only † ↓ global MDHAQ vs. advice only † ↓ fatigue vs. advice only † ↑ VAS Pain most affected joint †
Toprak-Celeny and Ozer, 2017 [73]	38 (100%)	No	20.6 (2.2)	Primary: BJHS *Excluded EDS*	Core	Body Weight, Resistance band (Weeks 3–8 only), Exercise Ball (Weeks 6–8 only)	8 weeks; 3×/week	↓ pain ↑ stability with eyes closed (static and dynamic)
Reychler et al., 2019 [74]	19 (100%)	Yes (10)	40.7 (14.1)	Primary: hEDS Secondary: reduced inspiratory muscle strength	IM	Breathing trainer	6 weeks; 5×/week	↑ 6MWT distance vs. baseline and control ↑ SNIP vs. baseline and control ↑ FEV1 vs. baseline and control
Daman et al., 2019 [75]	24 (100%)	No	22.0 (1.9)	Primary: JHS *Excluded regular exercisers/* *athletes*	LE	Body weight	4 weeks; 3×/week	↑ joint position sense ↓ pain ↑ quality of life
To and Alexander, 2019 [49]	102 (16%)	Yes (26)	34.9 (10.4)	Primary: JHS, GJH Secondary: Anterior knee pain (limited) *(Primary diagnosis for control)*	LE	*Not specified* but suspected to be body weight	16 weeks; 3×/week	↑ strength, all groups ↑ torque, all groups ↓ pain, all groups ↔ rate of strength gain between groups
Liaghat et al., 2020 [76]	12 (92%)	No	39.3 (13.9)	Primary: hEDS/HSD Secondary: Shoulder pain/ dislocations/ atraumatic instability	UE	Free weights	16 weeks; 3×/week	↑ self-report shoulder stability † ↓ pain † ↓ fatigue † ↑ isometric strength † ↓ proprioception error †
Luder et al., 2021 [77]	51 (100%)	Yes (24)	26.5 (4.5)	Primary: GJH (hEDS/HSD included in sample) *Excluded regular exercisers (4+ h/week)*	LE Core	*Not specified* but suspected to be free weights and/or machines as reference to 1RM and %1RM are made	12 weeks; 2×/week	↔ knee flexor strength ↔ knee extensor strength ↔ muscle CSA ↔ muscle mass ↔ muscle density

Age is reported as Mean (SD). * Median, not mean, due to non-parametric reporting. No IQR reported. † Pilot/Feasibility study; *p*-values not reported. JHS = Joint Hypermobility Syndrome; BJHS = Benign Joint Hypermobility Syndrome; EDS-HT = Ehlers–Danlos Syndrome, Hypermobility Type; hEDS =Hypermobility Ehlers–Danlos Syndrome; HSD = Hypermobility Spectrum Disorder; IM = Inspiratory Muscles; LE = Lower Extremity; MSK = Musculoskeletal; UE = Upper Extremity; WB = Whole Body; ↑ = statistically significant increase was noted; ↓ = statistically significant decrease was noted; ↔ = no change was noted.

**Table 4:** Summary of Recommendations for Future Research.

Identify and validate specific outcome measures that can be used in the hEDS/HSD population to enhance comparison between studies.
Conduct more research on children and adolescents utilizing control groups.
Identify proper progression of exercises and modes in adults and report ample detail on the exercises and modes being employed to allow for replication and implementation.
Investigate a proactive exercise prescription that can be recommended upon diagnosis.
Explore the relationship between baseline physical fitness and response to training programs in the hEDS/HSD population.
